# Socioeconomic deprivation is associated with reduced response and lower treatment persistence with TNF inhibitors in rheumatoid arthritis

**DOI:** 10.1093/rheumatology/kead261

**Published:** 2023-06-02

**Authors:** Sizheng Steven Zhao, Kira Rogers, Lianne Kearsley-Fleet, Kath Watson, Ailsa Bosworth, James Galloway, Suzanne Verstappen, Darren Plant, BSRBR-RA Contributors Group, BRAGGSS Collaborators, H Gaston, D Mulherin, T Price, T Sheeran, V Chalam, S Baskar, P Emery, A Morgan, M Buch, S Bingham, S O&hx2019;Reilly, L Badcock, M Regan, T Ding, C Deighton, G Summers, N Raj, R Stevens, N Williams, J Isaacs, P Platt, D Walker, L Kay, B Griffiths, W -F Ng, P Peterson, A Lorenzi, H Foster, M Friswell, B Thompson, M Lee, I Griffiths, A Hassell, P Dawes, C Dowson, S Kamath, J Packham, M Shadforth, A Brownfield, R Williams, C Mukhtyar, B Harrison, N Snowden, S Naz, J Ledingham, R Hull, F McCrae, A Thomas, S Young Min, R Shaban, E Wong, C Kelly, C Heycock, J Hamilton, V Saravanan, G Wilson, D Bax, L Dunkley, M Akil, R Tattersall, R Kilding, S Till, J Boulton, T Tait, M Bukhari, J Halsey, L Ottewell, C Buckley, D Situnayake, D Carruthers, K Grindulis, F Khatack, S Elamanchi, K Raza, A Filer, R Jubb, R Abernathy, M Plant, S Pathare, F Clarke, S Tuck, J Fordham, A Paul, M Bridges, A Hakim, D O&hx2019;Reilly, V Rajagopal, S Bhagat, C Edwards, P Prouse, R Moitra, D Shawe, A Bamji, P Klimiuk, A Bowden, W Mitchell, I Bruce, A Barton, R Gorodkin, P Ho, K Hyrich, W Dixon, A Rai, G Kitas, N Erb, R Klocke, K Douglas, A Pace, R Sandhu, A Whallett, F Birrell, M Allen, K Chaudhuri, C Chattopadhyay, J McHale, A Jones, A Gupta, I Pande, I Gaywood, P Lanyon, P Courtney, M Doherty, H Chinoy, T O&hx0027;Neill, A Herrick, A Jones, R Cooper, R Bucknall, C Marguerie, S Rigby, N Dunn, S Green, A Al-Ansari, S Webber, N Hopkinson, C Dunne, B Quilty, B Szebenyi, M Green, M Quinn, A Isdale, A Brown, B Saleem, A Samanta, P Sheldon, W Hassan, J Francis, A Kinder, R Neame, A Moorthy, W Al-Allaf, A Taggart, K Fairburn, F McKenna, M Green, A Gough, C Lawson, M Piper, E Korendowych, T Jenkinson, R Sengupta, A Bhalla, N McHugh, D Bond, R Luqmani, B Bowness, P Wordsworth, J David, W Smith, D Mewar, E Tunn, K Nelson, T Kennedy, J Nixon, A Woolf, M Davis, D Hutchinson, A Endean, D Coady, D Wright, C Morley, G Raftery, C Bracewell, L Kidd, I Abbas, C Filer, G Kallarackal, Anne Barton, Kimme L Hyrich, Jenny H Humphreys

**Affiliations:** Centre for Epidemiology Versus Arthritis, Division of Musculoskeletal and Dermatological Sciences, School of Biological Sciences, Faculty of Biology Medicine and Health, The University of Manchester, Manchester Academic Health Science Centre, Manchester, UK; Manchester Medical School, The University of Manchester, Manchester, UK; Centre for Epidemiology Versus Arthritis, Division of Musculoskeletal and Dermatological Sciences, School of Biological Sciences, Faculty of Biology Medicine and Health, The University of Manchester, Manchester Academic Health Science Centre, Manchester, UK; Centre for Epidemiology Versus Arthritis, Division of Musculoskeletal and Dermatological Sciences, School of Biological Sciences, Faculty of Biology Medicine and Health, The University of Manchester, Manchester Academic Health Science Centre, Manchester, UK; National Rheumatoid Arthritis Society (NRAS), Maidenhead, UK; Centre of Rheumatic Diseases, School of Immunology & Microbial Sciences, King’s College London, London, UK; Centre for Epidemiology Versus Arthritis, Division of Musculoskeletal and Dermatological Sciences, School of Biological Sciences, Faculty of Biology Medicine and Health, The University of Manchester, Manchester Academic Health Science Centre, Manchester, UK; Centre for Genetics and Genomics Versus Arthritis, Division of Musculoskeletal and Dermatological Sciences, School of Biological Sciences, Faculty of Biology Medicine and Health, The University of Manchester, Manchester Academic Health Science Centre, Manchester, UK; Centre for Genetics and Genomics Versus Arthritis, Division of Musculoskeletal and Dermatological Sciences, School of Biological Sciences, Faculty of Biology Medicine and Health, The University of Manchester, Manchester Academic Health Science Centre, Manchester, UK; NIHR Manchester Biomedical Research Centre, Manchester University NHS Foundation Trust, Manchester, UK; Centre for Epidemiology Versus Arthritis, Division of Musculoskeletal and Dermatological Sciences, School of Biological Sciences, Faculty of Biology Medicine and Health, The University of Manchester, Manchester Academic Health Science Centre, Manchester, UK; NIHR Manchester Biomedical Research Centre, Manchester University NHS Foundation Trust, Manchester, UK; Centre for Epidemiology Versus Arthritis, Division of Musculoskeletal and Dermatological Sciences, School of Biological Sciences, Faculty of Biology Medicine and Health, The University of Manchester, Manchester Academic Health Science Centre, Manchester, UK; NIHR Manchester Biomedical Research Centre, Manchester University NHS Foundation Trust, Manchester, UK

**Keywords:** deprivation, socioeconomic position, index of multiple deprivation, treatment response, RA

## Abstract

**Objective:**

To investigate the association between socioeconomic deprivation and outcomes following TNF inhibitor (TNFi) treatment.

**Methods:**

Individuals commencing their first TNFi in the British Society for Rheumatology Biologics Register for RA (BSRBR-RA) and Biologics in RA Genetics and Genomics Study Syndicate (BRAGGSS) cohort were included. Socioeconomic deprivation was proxied using the Index of Multiple Deprivation and categorized as 20% most deprived, middle 40% or 40% least deprived. DAS28-derived outcomes at 6 months (BSRBR-RA) and 3 months (BRAGGSS) were compared using regression models with the least deprived as referent. Risks of all-cause and cause-specific drug discontinuation were compared using Cox models in the BSRBR-RA. Additional analyses adjusted for lifestyle factors (e.g. smoking, BMI) as potential mediators.

**Results:**

16 085 individuals in the BSRBR-RA were included (mean age 56 years, 76% female), of whom 18%, 41% and 41% were in the most, middle and least deprived groups, respectively. Of 3459 included in BRAGGSS (mean age 57, 77% female), proportions were 22%, 36% and 41%, respectively. The most deprived group had 0.3-unit higher 6-month DAS28 (95% CI 0.22, 0.37) and were less likely to achieve low disease activity (odds ratio [OR] 0.76; 95% CI 0.68, 0.84) in unadjusted models. Results were similar for 3-month DAS28 (β = 0.23; 95% CI 0.11, 0.36) and low disease activity (OR 0.77; 95% CI 0.63, 0.94). The most deprived were more likely to discontinue treatment (hazard ratio 1.18; 95% CI 1.12, 1.25), driven by ineffectiveness rather than adverse events. Adjusted estimates were generally attenuated.

**Conclusion:**

Socioeconomic deprivation is associated with reduced response to TNFi. Improvements in determinants of health other than lifestyle factors are needed to address socioeconomic inequities.

Rheumatology key messages

- Socioeconomic deprivation is associated with reduced response to TNFi with or without adjusting for lifestyle factors.
- The most deprived group is more likely to discontinue treatment due to ineffectiveness than adverse events.
- Wider determinants of health, beyond lifestyle factors, may need to be addressed to reduce inequities.

## Introduction

Socioeconomic position has a well-recognized impact on health outcomes [1], which may be through individual behaviours, access to healthcare or wider determinants of health (e.g. education, job type or housing). Some factors closely related to socioeconomic deprivation have been studied in detail as risk or prognostic factors in RA, such as smoking and obesity [2, 3]. How such risk factors fit into the wider context of deprivation is unclear, for example whether the observed differences in RA outcomes would disappear if it were possible to eliminate smoking and obesity.

Greater focus on lifestyle interventions rather than socioeconomic deprivation may be partly due to the idea that common proxies (e.g. residential area or educational attainment) are not in themselves easily modifiable [1]. Socioeconomic inequity embodies a multitude of factors that can be intervened upon, including those that pertain to the health provider; for example, behaviours and biases among clinicians, or systems and structures at institutional levels that contribute to inequity. Recent global health crises have highlighted the importance and multifaceted nature of socioeconomic inequities and their impact on health provision and outcomes [4].

Better characterizing the associations between socioeconomic deprivation and treatment outcomes in RA would help to identify unmet needs in access to care as well as potential areas for intervention for both patients and providers. This analysis aimed to describe the association between socioeconomic position—measured using the Index of Multiple Deprivation (IMD)—and patient and disease characteristics and treatment outcomes following TNF inhibitor (TNFi) treatment in RA.

## Methods

This analysis used data from two prospective studies: the British Society for Rheumatology Biologics Register for RA (BSRBR-RA) and the Biologics in RA Genetics and Genomics Study Syndicate (BRAGGSS) cohort.

The BSRBR-RA is a national prospective observational study recruiting adults since 2001 with physician diagnosed RA at point of starting a biologic (b) and/or targeted synthetic (ts) DMARD [5]. Data are extracted from the medical record by local rheumatology teams at baseline (start of treatment), 6-monthly for the first 3 years, then annually thereafter. Baseline data included age, sex, BMI, smoking status (ever or never), age at diagnosis, rheumatoid factor status; measures of disease severity, namely the 28-joint disease activity score (DAS28) and components, HAQ, 36-Item Short Form Survey (SF36)-derived Mental and Physical Components Scores (MCS and PCS; from 2001 to 2006) [6], EuroQol-5 Dimension (EQ5D) and EQ-VAS (both 2006 onwards) [7]; and comorbidities, namely hypertension, ischaemic heart disease, stroke, respiratory disease (asthma and COPD), peptic ulcer disease, renal disease, diabetes and depression. DAS28 could be calculated by the recruiting site using ESR (mm/h) or CRP (mg/l) (ESR was more commonly used). The primary inflammatory marker of interest was ESR, which had more complete data than CRP. The current analysis used a data cut-off of 30 November 2020.

The BRAGGSS recruited patients from 60 centres across England since 2010. Assessments were performed at baseline, 3, 6 and 12 months, which allows additional insight into early treatment response. Baseline data comprised age, sex, rheumatoid factor status, disease duration, BMI, smoking status, HAQ, comorbidities (analysed as any or none) and DAS28 components. CRP was the primary inflammatory marker of interest in BRAGGSS analyses. DAS28 was primarily calculated using CRP in BRAGGSS and, where unavailable, using ESR. If participants had been recruited to both BSRBR-RA and BRAGGSS, then they were excluded from the BSRBR-RA dataset.

This analysis was limited to participants commencing their first TNFi. Ethical approval was obtained for BSRBR-RA (MREC 00/8/53) and BRAGGSS (COREC 04/Q1403/37); all participants provided written informed consent.

### Exposure

The ‘exposure’ of interest was IMD, a measure of relative deprivation of small geographic areas (or ‘neighbourhoods’) used by the UK Ministry of Housing, Communities and Local Government since the 1970s, which maps to an individual’s residential postcode. Deprivation in this context relates to lacking resources not limited to income, which distinguishes it from poverty. Full descriptions of IMD are provided in references [8–10]; in brief, it combines data from seven domains (income; employment; health deprivation and disability; education, skills and training; crime; barriers to housing and services; living environment) into one weighted score. This relative score is then used to rank neighbourhoods separately for each devolved nation of the UK. Each participant was assigned their rank score using their residential postcode at the time of registration using the most up to date ranking available, i.e. 2019 data for England, 2014 for Wales and 2012 for Scotland. Ranks were then mapped onto nationally determined quintiles separately for England, Wales and Scotland before combining; rank data were not available for Northern Ireland. To facilitate interpretation, analyses were performed using quintile 1 (top 20% most deprived), 2 and 3 (middle 40%), 4 and 5 (40% least deprived); these thresholds were chosen because IMD weights were designed to give greater distinction within the most deprived areas with lower granularity for more affluent areas [11].

### Outcomes

The following outcomes at 6 months were assessed in the BSRBR-RA: continuous variables comprising DAS28 composite score and components (i.e. swollen and tender joint counts, patient global [0–100 mm VAS] and ESR [mm/h]); and categorical variables comprising remission (DAS28 ≤ 2.6), low disease activity (LDA; ≤3.2) and EULAR response (good, moderate, no response). DAS28 and components at 6 months were analysed regardless of treatment discontinuation (i.e. intention-to-treat analysis). For categorical outcomes, participants who stopped treatment before month 6 were considered as non-responders. Time to treatment discontinuation (or ‘drug survival’) was also examined in the BSRBR-RA, including all-cause discontinuation and discontinuation due to ineffectiveness and adverse events.

### Statistics

Participant characteristics were compared across the three IMD groups. To facilitate comparison of variables with different scales, standardized difference were calculated between the 20% most deprived *vs* all remaining groups combined. The Kaplan–Meier estimator was used to compare drug survival (restricted to the first 5 years). Logistic regression models were used for LDA and remission, ordinal logistic regression for EULAR response and Cox proportional hazard models for drug survival, with the ‘40% least deprived’ group as referent. To estimate differences in 6-month DAS28 and components (or 3-month data in BRAGGSS), linear regression was used with 6-month (3-month in BRAGGSS) values as the outcome adjusted for baseline values (which in effect examines change in these variables over time without methodological limitations of using change scores [12]).

The precise causal relationships between IMD and baseline factors such as disease severity, comorbidities, smoking and BMI are unclear. General socioeconomic deprivation is more likely a cause of these factors, but these factors (which likely precede baseline assessment) may also influence where individuals live. Since their role as potential confounders (common causes of deprivation and treatment outcome) or mediators (causal intermediates) is unclear when using IMD as the proxy for deprivation, formal mediation analyses were not performed. Estimates were first presented from unadjusted models, and then adjusting for age, gender, baseline DAS28 (or DAS28 components in respective models), BMI, HAQ, number of comorbidities (categorized as 0, 1, 2, ≥3 in BSRBR-RA; as 0 or 1 in BRAGGSS), age at diagnosis, ever smoker status, rheumatoid factor status and calendar year of TNFi initiation. Presenting both adjusted and unadjusted estimates provides some insight into potential reverse causation and the direct effect of deprivation on treatment outcomes (i.e. effects not through potential mediators such as smoking or comorbidity).

For time-to-event analyses, drug survival was defined as duration between start and stop dates. Patients were censored from the analysis at their last follow-up date if still on that drug at that time. Separate analyses were performed for all-cause discontinuation and discontinuation due to ineffectiveness and adverse events.

Missing follow-up data on DAS28 or components were replaced with the next assessment; i.e. missing 6-month data in the BSRBR-RA replaced with 12-month, and missing 3-month BRAGGSS data replaced with 6-month. For all models, multiple imputation was used to account for missing baseline and outcome data (when outcomes were missing despite replacing with the subsequent follow-up assessment), using chained equations (30 imputed sets) and regression methods including: IMD categories, age, gender, baseline DAS28, BMI, comorbidities, age at diagnosis, ever smoker status, rheumatoid factor status, HAQ (only in the BSRBR-RA analysis) and DAS28 components. Imputed follow-up DAS28 was used to derive categorical outcome variables. Analyses were performed using Stata v14 (StataCorp, College Station, TX, USA).

## Results

### BSRBR-RA

Of 17 886 individuals with RA who started their first TNFi, 16 085 (90%) were eligible for analysis after excluding those with missing IMD data (*n* = 769) and/or were recruited to BRAGGSS (*n* = 1056) (flow chart in Supplementary Figure S1, available at *Rheumatology* online). There were no meaningful differences in characteristics between those included and excluded (Supplementary Table S1, available at *Rheumatology* online).

Baseline characteristics compared across IMD categories are shown in Table 1. Participants from the 20% most deprived areas were more frequently ever smokers and RF positive. Non-white ethnicity was more frequent in the most compared with least deprived areas (7 *vs* 3%). Missing data proportions were generally similar across IMD categories, except greater proportions of missing ethnicity and SF36 data in the 20% most deprived (Supplementary Table S2, available at *Rheumatology* online).

**Table 1.** Baseline characteristics compared across groups of socioeconomic deprivation in the BSRBR-RA

	All participants	20% most deprived	Middle 40%	40% least deprived	*P*-value	SMD^a^
*n*	16 085	2764	6707	6614		
Age, mean (s.d.), years	56.4 (12.4)	54.6 (12.3)	56.1 (12.4)	57.3 (12.3)	<0.001	−0.19
Female, *n* (%)	12 220 (76)	2091 (76)	4974 (75)	5155 (77)	0.075	−0.02
White ethnicity, *n* (%)	12 019 (95)	1874 (93)	4859 (95)	5286 (97)	<0.001	−0.13
RF positive, *n* (%)	9908 (64)	1753 (67)	4107 (65)	4048 (63)	0.003	−0.05
Age at diagnosis, mean (s.d.), years	44.5 (13.8)	43.6 (13.4)	44.2 (13.8)	45.2 (14.0)	<0.001	−0.08
BMI, mean (s.d.)	27.4 (7.8)	28.0 (8.1)	27.5 (7.9)	27.0 (7.4)	<0.001	−0.09
Ever smoker, *n* (%)	9243 (60)	1783 (67)	3902 (61)	3558 (55)	<0.001	−0.17
DAS28, mean (s.d.)	6.3 (1.1)	6.3 (1.1)	6.3 (1.1)	6.3 (1.1)	0.029	−0.17
Tender joint count, mean (s.d.)	14.8 (7.5)	14.8 (7.6)	15.0 (7.5)	14.6 (7.4)	0.011	−0.11
Swollen joint count, mean (s.d.)	10.4 (6.1)	10.0 (6.1)	10.4 (6.1)	10.5 (6.1)	0.001	−0.04
ESR, median (IQR), mm/h	36.0 (20.0, 59.0)	36.0 (20.0, 59.0)	37.0 (21.0, 60.0)	36.0 (20.0, 58.0)	0.026	−0.05
CRP, median (IQR), mg/l	23.0 (9.0, 50.0)	23.0 (10.0, 50.0)	22.0 (9.0, 50.0)	23.0 (9.0, 51.0)	0.76	−0.07
Patient global VAS, mean (s.d.), mm	72.1 (20.3)	73.1 (20.0)	71.9 (20.5)	71.8 (20.3)	0.013	−0.19
HAQ, mean (s.d.)	1.9 (0.7)	2.0 (0.6)	1.9 (0.7)	1.8 (0.7)	<0.001	−0.23
SF36 Physical Component Score, mean (s.d.)	16.3 (8.6)	15.8 (8.6)	16.1 (8.5)	16.7 (8.7)	<0.001	−0.22
SF36 Mental Component Score, mean (s.d.)	42.2 (11.7)	39.9 (11.3)	42.0 (11.8)	43.3 (11.7)	<0.001	−0.29
EQ5D, median (IQR)	0.5 (0.1, 0.7)	0.5 (0.0, 0.6)	0.6 (0.1, 0.7)	0.6 (0.1, 0.7)	<0.001	−0.32
EQ-VAS, mean (s.d.)	52.3 (23.1)	49.7 (22.6)	52.0 (23.3)	53.7 (23.0)	<0.001	−0.13
Comorbities, *n* (%)						
No comorbidities	6502 (40)	967 (35)	2665 (40)	2870 (43)	<0.001	−0.13
1 comorbidity	4731 (29)	829 (30)	1939 (29)	1963 (29)		
2 comorbidities	2736 (17)	522 (19)	1129 (17)	1085 (16)		
≥3 comorbidities	2116 (13)	446 (16)	881 (13)	789 (12)		
Hypertension, *n* (%)	4636 (29)	816 (30)	1908 (29)	1912 (29)	0.61	−0.01
Ischaemic heart disease, *n* (%)	898 (6)	206 (7)	399 (6)	293 (4)	<0.001	−0.09
Stroke, *n* (%)	331 (2)	63 (2)	151 (2)	117 (2)	0.061	−0.03
Respiratory disease, *n* (%)	2348 (15)	494 (18)	950 (14)	904 (13)	<0.001	−0.09
Peptic ulcer disease, *n* (%)	1077 (7)	193 (7)	446 (7)	438 (7)	0.71	−0.01
Renal disease, *n* (%)	372 (2)	75 (3)	150 (2)	147 (2)	0.29	−0.02
Diabetes, *n* (%)	981 (6)	199 (7)	426 (6)	356 (5)	<0.001	−0.06
Depression, *n* (%)	3100 (19)	689 (25)	1287 (19)	1124 (17)	<0.001	−0.14

a Standardized mean difference compared between 20% most deprived *vs* all other participants. BSRBR-RA: British Society for Rheumatology Biologics Register for RA; DAS28: 28-joint disease activity score; EQ5D: EuroQol-5 Dimension questionnaire; EQ-VAS: EuroQol visual analogue scale; IQR: interquartile range; SF36: 36-Item Short Form Survey; VAS: visual analogue scale.

Tender joint count and patient global score showed greater standardized difference between 20% most deprived and others, compared with other DAS28 components. The largest standardized differences were observed for HAQ, PCS, MCS and EQ5D, indicating disparity in functional impairment and lower quality of life between the most deprived and the remaining deprivation groups.

Comorbidity burden was higher in individuals from more deprived areas; most notably, depression was more common in the most *vs* least deprived areas (25 *vs* 17%).

### Treatment response

**Figure 1.**
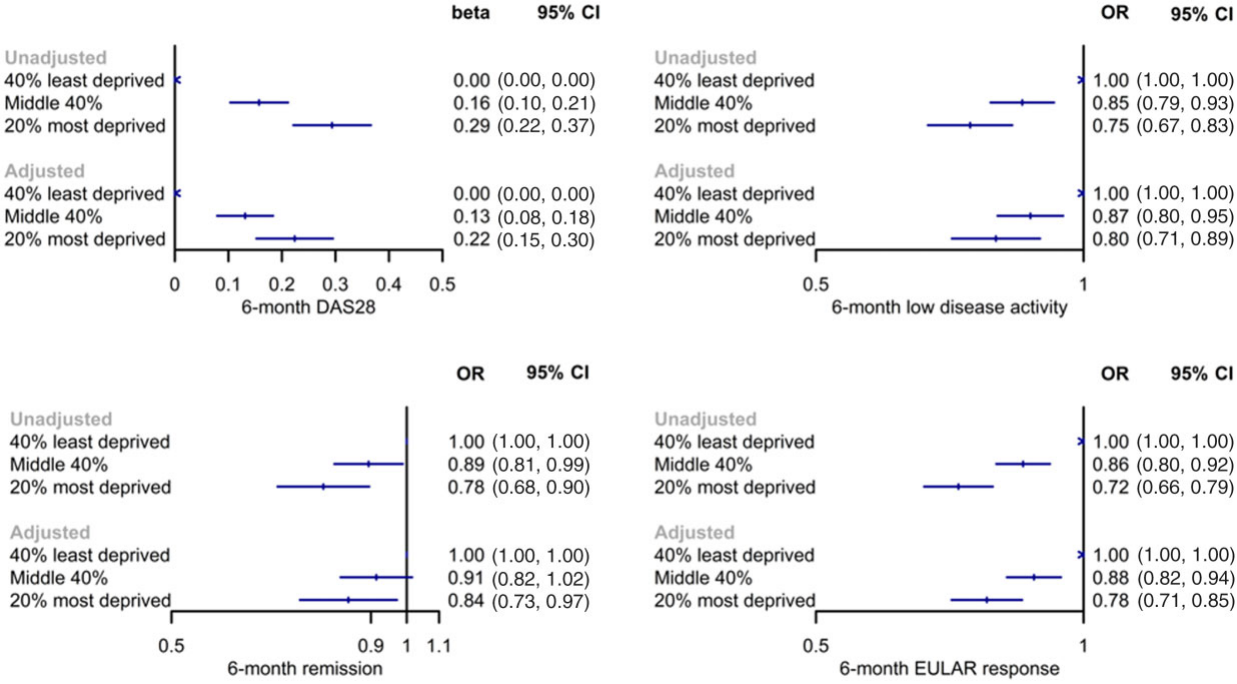
Models comparing 6-month treatment response across groups of socioeconomic deprivation in BSRBR-RA. BSRBR-RA: British Society for Rheumatology Biologics Register for RA; DAS28: 28-joint disease activity score; OR: odds ratio

A total of 15 830 participants were included in the treatment outcome analysis after excluding those with incomplete data for drug start or stop dates. Proportion of missing baseline and 6-month DAS28 and components and imputed values at baseline and 6 months are shown in Supplementary Tables S3 and S4, available at *Rheumatology* online. Compared with the least deprived group, increasing deprivation was associated with higher DAS28 at 6 months (Figure 1). In the unadjusted model, 6-month DAS28 was 0.30 units higher in the most deprived group and 0.17 units higher in the middle 40%. Adjusted estimates were numerically similar.

Increasing socioeconomic deprivation was also associated with lower odds of achieving LDA, remission and EULAR response in unadjusted models (Figure 1). For example, the most deprived group had 24% lower odds of achieving LDA (95% CI 0.68, 0.84) and 11% lower odds of achieving remission (95% CI 0.81, 0.98) in unadjusted models. Adjusted models provided concordant results, except for remission where estimates included the null.

All DAS28 components were higher at 6 months with increasing levels of socioeconomic deprivation (Supplementary Figure S2, available at *Rheumatology* online). However, the lesser response in 6-month DAS28 outcomes appears to be driven more so by tender joint count than swollen joint count; for example, tender joint count was 1.2 units higher in the most *vs* least deprived, whereas the equivalent estimate for swollen joint count was 0.5 units.

### Drug survival

**Figure 2.**
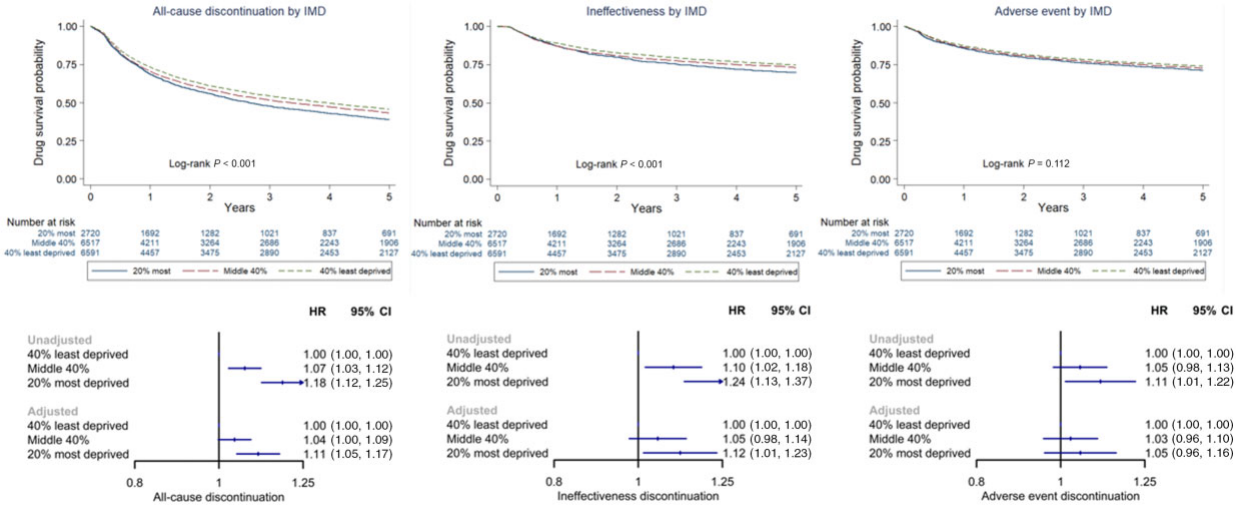
Probability of drug discontinuation compared across groups of socioeconomic deprivation in BSRBR-RA. BSRBR-RA: British Society for Rheumatology Biologics Register for RA; HR: hazard ratio; IMD: Index of Multiple Deprivation

The overall analysis population (*n* = 16 085) was used for drug survival analyses. Compared with the least deprived group, participants in the more deprived subgroups had increasing probability of all-cause drug discontinuation (Figure 2). This appears to be driven by discontinuation due to ineffectiveness rather than due to adverse events (Figure 2). The median time to all-cause treatment discontinuation was shortest in the most deprived group (2.6 years *vs* 3.8 years in the least deprived group), with correspondingly higher probability of stopping TNFi at 1 year compared with other socioeconomic groups (Table 2).

**Table 2.** Median time to drug discontinuation and probability of remaining on drug in the BSRBR-RA

	20% most deprived	Middle 40%	40% least deprived
Time to any-cause discontinuation, median (IQR), years	2.6 (2.4, 2.9)	3.3 (3.0, 3.6)	3.8 (3.6, 4.1)
Probability remaining on drug, % (95% CI)			
Year 1	68.5 (66.7, 70.3)	70.5 (69.3, 71.6)	73.4 (72.3, 74.5)
Year 3	47.6 (45.6, 49.5)	51.6 (50.3, 52.9)	54.3 (53.0, 55.5)
Year 5	38.8 (36.8, 40.8)	43.1 (41.8, 44.4)	45.6 (44.3, 46.9)

BSRBR-RA: British Society for Rheumatology Biologics Register for RA. IQR: interquartile range.

Compared with the least deprived group, the hazard of all-cause discontinuation was HR 1.18 higher (95% CI 1.12, 1.25) in the most deprived group, and HR 1.07 higher (95% CI 1.03, 1.12) in the middle 40% in unadjusted models. Results for cause-specific discontinuation are shown in Figure 2. Adjusted models provided similar estimates.

### BRAGGSS

Of 3584 participants starting TNFi, 3459 had IMD data to allow analysis. Excluded individuals were older (49.7 *vs* 47.1 years) and more often male (31 *vs* 23%) than included individuals, but there were no other meaningful differences in characteristics (Supplementary Table S5, available at *Rheumatology* online).

Baseline characteristics compared across IMD categories are shown in Table 3, with similar proportions of missing data except for higher proportion of missing HAQ in the most deprived group (Supplementary Table S6, available at *Rheumatology* online). Participants from the most deprived areas were younger at diagnosis and treatment initiation, had higher BMI and HAQ, and were more frequently ever smokers. Patient global score showed greater standardized difference between most deprived and other groups, compared to other DAS28 components.

**Table 3.** Baseline characteristics compared across groups of socioeconomic deprivation in BRAGGSS

	All participants	20% most deprived	Middle 40%	40% least deprived	*P*-value	SMD^a^
*n*	3459	770	1256	1433		
Age, mean (s.d.), years	57.3 (12.4)	55.5 (11.3)	56.7 (12.7)	58.7 (12.4)	<0.001	−0.19
Female, *n* (%)	2652 (77%)	589 (77%)	976 (78%)	1087 (76%)	0.50	−0.004
RF positive, *n* (%)	1961 (68%)	461 (71%)	688 (66%)	812 (69%)	0.057	0.08
Age at diagnosis, mean (s.d.), years	47.1 (13.7)	45.9 (12.6)	46.7 (13.7)	48.1 (14.2)	0.001	−0.12
BMI, mean (s.d.), kg/m^2^	29.3 (13.4)	31.4 (22.3)	28.7 (7.1)	28.6 (10.4)	<0.001	0.16
Ever smoker, *n* (%)	1671 (61%)	446 (70%)	605 (61%)	620 (56%)	<0.001	0.25
DAS28, mean (s.d.)	5.7 (0.9)	5.7 (0.9)	5.7 (0.9)	5.7 (0.9)	0.74	−0.003
Tender joint count, mean (s.d.)	14.5 (7.0)	14.4 (7.1)	14.6 (6.9)	14.5 (6.9)	0.81	0.04
Swollen joint count, mean (s.d.)	8.3 (5.2)	7.6 (4.7)	8.2 (4.9)	8.8 (5.6)	<0.001	−0.08
ESR, median (IQR), mm/h	25.0 (12.0, 42.0)	27.0 (13.0, 44.0)	25.0 (12.0, 44.0)	23.0 (12.0, 40.0)	0.015	0.08
CRP, median (IQR), mg/l	10.3 (3.7, 25.8)	11.4 (4.3, 26.5)	9.9 (3.6, 24.6)	9.9 (3.6, 25.8)	0.070	0.08
Patient global VAS, mean (s.d.)	73.2 (18.6)	75.0 (18.1)	71.8 (19.3)	73.5 (18.2)	0.001	0.16
HAQ, mean (s.d.)	1.7 (0.7)	1.9 (0.6)	1.7 (0.7)	1.6 (0.7)	<0.001	0.30
Any comorbidity, *n* (%)	823 (24%)	201 (26%)	321 (26%)	301 (21%)	0.005	0.04

a Standardized mean difference compared between 20% most deprived *vs* all other participants. BRAGGSS: Biologics in RA Genetics and Genomics Study Syndicate; DAS28: 28-joint disease activity score; IQR: interquartile range; VAS: visual analogue scale.

**Figure 3.**
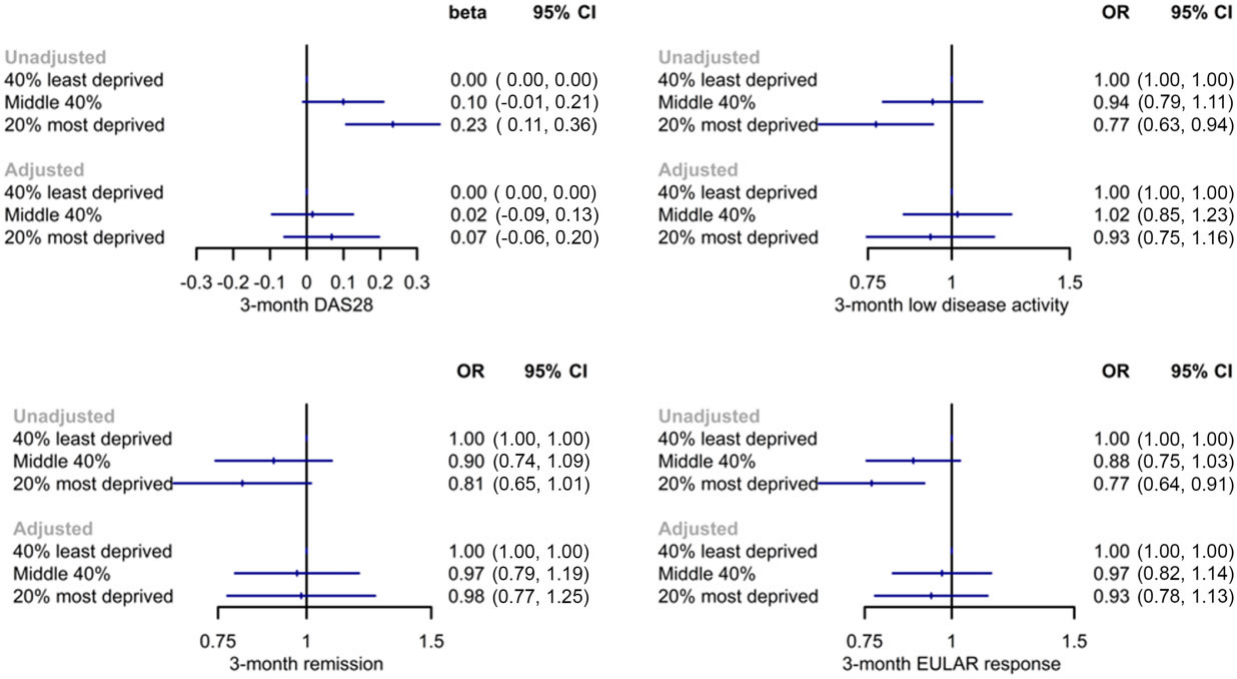
Models comparing 3-month treatment response across groups of socioeconomic deprivation in BRAGGSS. BRAGGSS: Biologics in RA Genetics and Genomics Study Syndicate; OR: odds ratio

Proportion of missing baseline and 3-month DAS28 and components and imputed values at baseline and 3 months are shown in Supplementary Tables S7 and S8, available at *Rheumatology* online. Compared with the least deprived group, increasing deprivation was associated with 0.23 units higher DAS28 at 3 months (95% CI 0.11, 0.36) (Figure 3). Adjusted estimates were attenuated to include the null.

Increasing socioeconomic deprivation was also associated with lower odds of achieving LDA, remission and EULAR response in unadjusted models (Figure 3). For example, the most deprived group had lower odds of achieving LDA (odds ratio 0.77; 95% CI 0.63, 0.94). Adjusted estimates all included the null.

Of the DAS28 components, tender joint count and patient global score showed the greatest 3-month difference across increasing levels of socioeconomic deprivation; by contrast, swollen joint count and CRP were not significantly different when compared with the least deprived (Supplementary Fig. S3, available at *Rheumatology* online).

## Discussion

This study showed that socioeconomic deprivation was associated with reduced response to TNFi and increased risk of treatment discontinuation. Median time to discontinuation differed by 1 year between the 20% most deprived and the 40% least deprived groups. The most deprived subgroup had lower odds of achieving remission, LDA or EULAR response compared with the least deprived. Reduced response to treatment, as measured using DAS28 and its components, in more deprived groups appears to be driving the greater risk of treatment discontinuation, whereas discontinuation due to adverse events was similar.

In analyses of the larger BSRBR-RA population, model estimates were generally unchanged after adjusting for potential lifestyle mediators of the effect of deprivation on treatment outcomes (e.g. smoking and BMI), suggesting that socioeconomic deprivation may influence treatment outcomes through paths other than smoking, BMI or comorbidity burden. Loss of statistical significance in adjusted models of remission is likely related to power, as the proportion achieving remission was small (and smaller than all other categorical outcomes). In the independent BRAGGSS analysis, the direction of 3-month estimates was similar and consistent with the larger BSRBR-RA data. However, formal mediation analysis is required to examine this in greater detail.

A recent systematic literature review of the effect of socioeconomic deprivation in RA found that the majority of studies reported cross-sectional associations between socioeconomic deprivation and more severe disease [13]. A smaller number of longitudinal studies, limited to conventional synthetic DMARD-treated cohorts, generally agreed that socioeconomic deprivation is associated with greater physical functional impairment over time. Only two studies investigated outcomes in biologic-treated cohorts, both in the context of difficult-to-treat RA [14, 15]. These studies found that lower socioeconomic position (proxied using educational attainment or IMD) was an independent predictor of developing difficult-to-treat RA (definitions of which included failure of multiple b/tsDMARDs). The current study is therefore the first to describe detailed treatment outcomes among a contemporary b/tsDMARD-treated RA cohort, to our knowledge.

There are several potential explanations for these findings. Socioeconomic position is closely associated with adverse lifestyle factors, such as smoking and raised BMI, which are associated with poorer treatment outcomes in RA [16]. Socioeconomic deprivation is also associated with mental and physical comorbidities [17, 18] that have been shown to influence treatment response [19, 20]. Prior research on overall mortality and cardiovascular diseases has estimated that lifestyle factors account for only 20–30% of the socioeconomic inequality in health outcomes [21]. Taken together, these findings suggest that focusing intervention on lifestyle factors alone will be insufficient to address disparate treatment outcomes due to socioeconomic inequity.

Other explanations for the association between socioeconomic position and treatment outcomes are the dynamic interaction between individuals’ social contexts and allocation of resources and configuration of healthcare services [22]. This may include physical access (e.g. to specialist centres or transport to hospitals), financial implications (e.g. reduced pay to attend appointments or transport costs), patient or clinician perception of benefit, or attitudes and biases of healthcare providers and institutions (e.g. inequality and discrimination relating to race [23]). Distribution of IMD quintiles in both BSRBR-RA and BRAGGSS closely matched that of the general population, which suggests representative recruitment from all socioeconomic groups. However, it remains unclear whether RA epidemiology and TNFi use differs across socioeconomic groups in the general population served by the recruiting sites. One limitation of the current study is the lack of ethnic diversity in the study population. Research from other health conditions has shown important interaction between ethnicity and socioeconomic status [24], that is, deprivation may disproportionately affect health outcomes in ethnic minorities. Socioeconomic deprivation is also associated with lower levels of education and health-literacy, such that individuals may not optimally utilize healthcare [25]. Discussions around these factors and wider determinants of health (e.g. poverty, housing or environment) are less common in the RA literature, yet inequalities in provision of care due to provider biases are modifiable. Results of this study suggest that deprivation has an important effect on health outcomes. Future research, both observational studies and clinical trials, should seek to measure deprivation more routinely and report it more transparently. Clinicians may consider potential for individual- and system-level biases. Further research is needed on the effect of clinician- or institution-level biases that affect treatment outcomes, including but not limited to ethnicity.

Standardized differences in baseline characteristics (which facilitate comparison despite differences in scale) revealed interesting insights. Compared with other DAS28 components, tender joints count and patient global scores (i.e. more subjective components) were more different between the 20% most deprived and the remainder. Similarly, the largest standardized differences in disease indices were observed for quality of life and physical function. These findings may reflect poorer general health independent of RA, which is supported by the higher burden of comorbid depression (largest standardized difference of the comorbidities) in the most deprived subgroup. Optimizing comorbidities is essential in the management of RA [19], and should be incorporated into treat-to-target approaches.

Prior research raised concerns that individuals with elevated DAS28 due to non-inflammatory symptoms (e.g. disproportionately high patient global [26, 27]) may not respond to TNFi that primarily target inflammatory pathology. Results of the current analysis suggest that the poorer response in 6-month DAS28 in the most deprived group may be driven by tender more than swollen joint count. Properties of the composite DAS28 were not the primary focus of this study, but should be a topic for future research.

A strength of this analysis is use of large real-world populations reflective of routine practice in the UK. Findings were concordant across a range of treatment outcome definitions. There were also limitations. IMD is an imperfect proxy for socioeconomic position, not least because it is a geographical rather than individual-level index. Although IMD captures several aspects of socioeconomic deprivation, the relative influence of each is not available for analyses. The temporal relationship between lifestyle factors and area of residence could not be established from this dataset; therefore, their role as confounders or mediators in the adjusted model is unclear. Whether the variables acted as confounders or mediators, the adjusted estimates would (typically) be attenuated. Similar unadjusted and adjusted estimates suggest that the role they play in either case is limited, and that other factors are needed to explain the observed association. Socioeconomic position can also influence health inequalities through specific characteristics (disability, ethnicity) or socially excluded groups (e.g. people experiencing homelessness) that are not captured in IMD. These factors and how they interact with each other [28] should be areas for future study. Lastly, the current analysis focused on TNFi; whether deprivation influences treatment outcomes of other drugs (e.g. those requiring hospital attendance for infusion) should be investigated in future studies.

To our knowledge, this is the first study to describe detailed treatment outcomes among a contemporary b/tsDMARD-treated RA cohort, finding that socioeconomic deprivation is associated with reduced response to TNFi and increased risk of treatment discontinuation. These associations were unchanged after adjusting for potential lifestyle mediators. This suggests efforts should also focus on addressing inequities of access to and provision of care to improve outcomes for patients across the socioeconomic spectrum.

## Supplementary data

Supplementary data are available at *Rheumatology* online.

## Supplementary Material

kead261_Supplementary_Data

## Data Availability

The data that support the findings of this study are available from the British Society for Rheumatology. Restrictions apply to the availability of these data (see: https://www.rheumatology.org.uk/practice-quality/registers/requesting-registers-data).
