# Supplementary material for: Socioeconomic deprivation is associated with reduced response and lower treatment persistence with TNF inhibitors in rheumatoid arthritis
Source: Rheumatology (Oxford). 2023 Jun 2;63(3):648–56. doi: 10.1093/rheumatology/kead261 (PMC10907806; doi:10.1093/rheumatology/kead261)

## Supplementary materials:

**Socioeconomic deprivation is associated with reduced response and lower treatment persistence with TNF inhibitors in rheumatoid arthritis**

Sizheng Steven Zhao*^1^, Kira Rogers*^2^, Lianne Kearsley-Fleet^1^, Kath Watson^1^, Ailsa Bosworth^3^, James Galloway^4^, Suzanne Verstappen^1^, Darren Plant^5^, BSRBR-RA Contributors Group, BRAGGSS, Anne Barton^5,6^, Kimme L Hyrich^1,6^, Jenny H Humphreys^1,6^

1. Centre for Epidemiology Versus Arthritis, Division of Musculoskeletal and Dermatological Sciences, School of Biological Sciences, Faculty of Biology Medicine and Health, The University of Manchester, Manchester Academic Health Science Centre, Manchester, United Kingdom

2. Manchester Medical School, The University of Manchester

3. National Rheumatoid Arthritis Society (NRAS), Maidenhead, UK

4. Centre of Rheumatic Diseases, School of Immunology & Microbial Sciences, King’s College London, London, UK

5. Centre for Genetics and Genomics Versus Arthritis, Division of Musculoskeletal and Dermatological Sciences, School of Biological Sciences, Faculty of Biology Medicine and Health, The University of Manchester, Manchester Academic Health Science Centre, Manchester, UK

6. NIHR Manchester Biomedical Research Centre, Manchester University NHS Foundation Trust, Manchester, United Kingdom.

*Zhao and Rogers contributed equally.

Contents

[Supplementary materials: 1](#_Toc125975065)

[Figure S1. Flow chart of the BSRBR-RA analysis population selection. 2](#_Toc125975066)

[Table S1. Characteristics of participants included and excluded from BSRBR-RA analysis due to missing IMD. 3](#_Toc125975067)

[Table S2. Missing data proportions in each group of socioeconomic deprivation in the BSRBR-RA 4](#_Toc125975068)

[Table S3. Proportion of missing baseline and 6-month DAS28 and components in the BSRBR-RA. 4](#_Toc125975069)

[Table S4. Imputed treatment response measures at baseline and 6 months in the BSRBR-RA. 5](#_Toc125975070)

[Figure S2. Models comparing 6-month DAS components across groups of socioeconomic deprivation in the BSRBR-RA. 6](#_Toc125975071)

[Table S5. Characteristics of participants included and excluded from analysis of BRAGGSS due to missing IMD. 7](#_Toc125975072)

[Table S6. Missing data proportions in each group of socioeconomic deprivation in BRAGGSS 7](#_Toc125975073)

[Table S7. Proportion of missing baseline and 6-month DAS28 and components in BRAGGSS. 8](#_Toc125975074)

[Table S8. Imputed treatment response measures at baseline and 6 months in BRAGGSS. 8](#_Toc125975075)

[Figure S3. Models comparing 3-month DAS components across groups of socioeconomic deprivation in BRAGGSS. 9](#_Toc125975076)

## Figure S1. Flow chart of the BSRBR-RA analysis population selection.

1,801 excluded for either:

missing IMD (n=769) and/or

recruited to BRAGGSS (n=1,056)

17,886

16,085 in overall analysis population

Missing drug start or stop dates (n=301)

15,830 in response analysis

## Table S1. Characteristics of participants included and excluded from BSRBR-RA analysis due to missing IMD.

|  | Excluded | Included | p-value |
| --- | --- | --- | --- |
| N | 1801 | 16085 |  |
| Age, mean (SD) | 56.7 (12.7) | 56.4 (12.4) | 0.28 |
| Female, n (%) | 1368 (76%) | 12220 (76%) | 0.99 |
| White ethnicity, n (%) | 1354 (98%) | 12019 (95%) | <0.001 |
| RF positive, n (%) | 984 (61%) | 9908 (64%) | 0.010 |
| Age at diagnosis, mean (SD) | 47.0 (13.9) | 44.5 (13.8) | <0.001 |
| BMI, mean (SD) | 28.3 (8.0) | 27.4 (7.8) | <0.001 |
| Ever smoked, n (%) | 1001 (59%) | 9243 (60%) | 0.67 |
| Baseline DAS28, mean (SD) | 6.2 (1.1) | 6.3 (1.1) | 0.007 |
| Tender joint count, mean (SD) | 14.7 (7.0) | 14.8 (7.5) | 0.48 |
| Swollen joint count, mean (SD) | 9.2 (5.4) | 10.4 (6.1) | <0.001 |
| ESR (mm/hr), median (IQR) | 28.0 (14.0, 48.0) | 36.0 (20.0, 59.0) | <0.001 |
| CRP (mg/l), median (IQR) | 12.0 (5.0, 31.7) | 23.0 (9.0, 50.0) | <0.001 |
| Patient global VAS, mean (SD) | 73.7 (18.9) | 72.1 (20.3) | 0.001 |
| HAQ, mean (SD) | 1.8 (0.7) | 1.9 (0.7) | <0.001 |
| SF36 Physical Component Score, mean (SD) | 16.9 (8.9) | 16.3 (8.6) | 0.19 |
| SF36 Mental Component Score, mean (SD) | 42.0 (12.0) | 42.2 (11.7) | 0.65 |
| EQ5D, median (IQR) | 0.5 (0.0, 0.7) | 0.5 (0.1, 0.7) | 0.048 |
| EQ-VAS, mean (SD) | 49.5 (22.2) | 52.3 (23.1) | <0.001 |
| No comorbidities, n (%) | 719 (40%) | 6502 (40%) | <0.001 |
| 1 comorbidity | 502 (28%) | 4731 (29%) |  |
| 2 comorbidities | 277 (15%) | 2736 (17%) |  |
| 3 comorbidities | 303 (17%) | 2116 (13%) |  |
| Hypertension, n (%) | 506 (28%) | 4636 (29%) | 0.52 |
| Ischaemic heart disease, n (%) | 86 (5%) | 898 (6%) | 0.15 |
| Stroke, n (%) | 27 (1%) | 331 (2%) | 0.11 |
| Respiratory disease, n (%) | 302 (17%) | 2348 (15%) | 0.014 |
| Peptic ulcer disease, n (%) | 100 (6%) | 1077 (7%) | 0.064 |
| Renal disease, n (%) | 38 (2%) | 372 (2%) | 0.59 |
| Diabetes, n (%) | 121 (7%) | 981 (6%) | 0.30 |
| Depression, n (%) | 365 (20%) | 3100 (19%) | 0.31 |
| RF, rheumatoid factor; HAQ, health assessment questionnaire; SF36, 36-Item Short Form Survey; EQ5D, EuroQol-5 Dimension questionnaire; EQ-VAS, EuroQol visual analogue scale. | | | |

## Table S2. Missing data proportions in each group of socioeconomic deprivation in the BSRBR-RA

|  | Overall | 20% most deprived | Middle 40% | 40% least deprived |
| --- | --- | --- | --- | --- |
| N | 16,085 | 2,764 | 6,614 | 6,707 |
| Ethnicity | 3492 (21.7) | 749 (27.1) | 1485 (22.5) | 1258 (18.8) |
| RF positive | 698 (4.3) | 131 (4.7) | 286 (4.3) | 281 (4.2) |
| Age at diagnosis | 236 (1.5) | 52 (1.9) | 97 (1.5) | 87 (1.3) |
| BMI | 2712 (16.9) | 499 (18.1) | 1099 (16.6) | 1114 (16.6) |
| Ever smoked | 584 (3.6) | 104 (3.8) | 246 (3.7) | 234 (3.5) |
| Baseline DAS28 | 105 (0.7) | 15 (0.5) | 50 (0.8) | 40 (0.6) |
| Tender joint count | 543 (3.4) | 95 (3.4) | 237 (3.6) | 211 (3.2) |
| Swollen joint count | 549 (3.4) | 96 (3.5) | 239 (3.6) | 214 (3.2) |
| ESR | 2344 (14.6) | 384 (13.9) | 983 (14.9) | 977 (14.6) |
| CRP | 9423 (58.6) | 1537 (55.6) | 3920 (59.3) | 3966 (59.1) |
| Patient global VAS | 736 (4.6) | 141 (5.1) | 316 (4.8) | 279 (4.2) |
| HAQ | 2275 (14.1) | 419 (15.2) | 922 (13.9) | 934 (13.9) |
| SF36 Physical Component Score | 7677 (47.7) | 1438 (52) | 3194 (48.3) | 3045 (45.4) |
| SF36 Mental Component Score | 7677 (47.7) | 1438 (52) | 3194 (48.3) | 3045 (45.4) |
| EQ5D | 10972 (68.2) | 1799 (65.1) | 4474 (67.6) | 4699 (70.1) |
| EQ-VAS | 10552 (65.6) | 1738 (62.9) | 4323 (65.4) | 4491 (67) |
| Data presented as number of patients with missing data (percentage). RF, rheumatoid factor; HAQ, health assessment questionnaire; SF36, 36-Item Short Form Survey; EQ5D, EuroQol-5 Dimension questionnaire; EQ-VAS, EuroQol visual analogue scale. | | | | |

## Table S3. Proportion of missing baseline and 6-month DAS28 and components in the BSRBR-RA.

|  |  | n | % |
| --- | --- | --- | --- |
| baseline | DAS28 | 102 | 0.6 |
|  | Tender joint count | 532 | 3.4 |
|  | Swollen joint count | 538 | 3.4 |
|  | ESR | 2,268 | 14.3 |
|  | Patient global score | 727 | 4.6 |
| 6 months | DAS28 | 2,496 | 15.8 |
|  | Tender joint count | 2,862 | 18.1 |
|  | Swollen joint count | 2,864 | 18.1 |
|  | ESR | 4,099 | 25.9 |
|  | Patient global score | 3,246 | 20.5 |
| DAS28 could be calculated using CRP or ESR as per the recruiting centre. | | | |

## Table S4. Imputed treatment response measures at baseline and 6 months in the BSRBR-RA.

|  |  | 20% most deprived | Middle 40% | 40% least deprived |
| --- | --- | --- | --- | --- |
| Percentage response at 6 months | Remission | 14.1% | 15.7% | 17.4% |
|  | Low disease activity | 24.5% | 26.7% | 30.2% |
|  | No EULAR response | 29.7% | 25.5% | 22.8% |
|  | Moderate EULAR response | 46.9% | 48.8% | 48.3% |
|  | Good EULAR response | 23.4% | 25.7% | 28.8% |
| Baseline | DAS28, mean | 6.31 | 6.32 | 6.28 |
|  | Tender joint count, mean | 14.7 | 15.0 | 14.6 |
|  | Swollen joint count, mean | 10.0 | 10.4 | 10.5 |
|  | ESR, mean, mm/hr | 73.0 | 71.7 | 71.6 |
|  | Patient global, mean | 40.3 | 40.5 | 39.1 |
| 6 months | DAS28, mean | 4.42 | 4.29 | 4.11 |
|  | Tender joint count, mean | 7.0 | 6.5 | 5.8 |
|  | Swollen joint count, mean | 4.3 | 4.2 | 4.0 |
|  | ESR, mean, mm/hr | 46.1 | 42.6 | 40.5 |
|  | Patient global, mean | 29.4 | 28.4 | 27.0 |

## Figure S2. Models comparing 6-month DAS components across groups of socioeconomic deprivation in the BSRBR-RA.


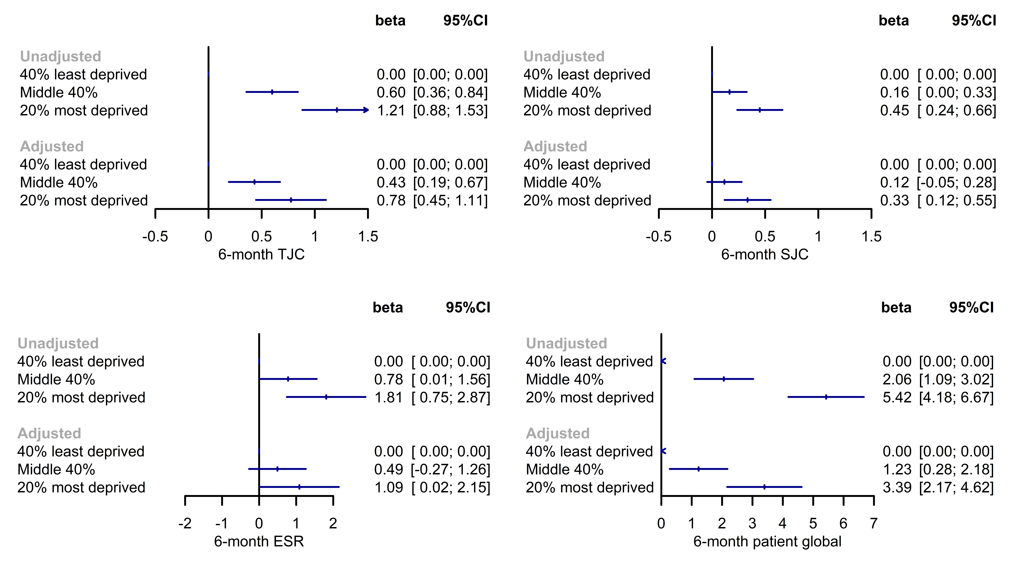


## Table S5. Characteristics of participants included and excluded from analysis of BRAGGSS due to missing IMD.

|  | Excluded | Included | p-value |
| --- | --- | --- | --- |
| N | 125 | 3459 |  |
| Age, mean (SD) | 57.6 (13.8) | 57.3 (12.4) | 0.77 |
| Female, n (%) | 81 (69%) | 2652 (77%) | 0.042 |
| RF positive, n (%) | 62 (63%) | 1961 (68%) | 0.24 |
| Age at diagnosis, mean (SD) | 49.7 (13.7) | 47.1 (13.7) | 0.048 |
| BMI, mean (SD) | 29.8 (7.1) | 29.3 (13.4) | 0.70 |
| Ever smoked, n (%) | 56 (56%) | 1671 (61%) | 0.35 |
| DAS28, mean (SD) | 5.7 (0.9) | 5.8 (0.8) | 0.55 |
| Tender joint count, mean (SD) | 14.4 (7.2) | 14.5 (7.0) | 0.86 |
| Swollen joint count, mean (SD) | 8.3 (5.0) | 8.3 (5.2) | 0.90 |
| ESR (mm/hr), median (IQR) | 23.0 (11.0, 39.0) | 25.0 (12.0, 42.0) | 0.59 |
| CRP (mg/l), median (IQR) | 9.9 (4.3, 31.7) | 10.3 (3.7, 25.8) | 0.46 |
| Patient global VAS, mean (SD) | 75.6 (16.7) | 73.2 (18.6) | 0.19 |
| HAQ, mean (SD) | 1.7 (0.7) | 1.7 (0.7) | 0.64 |
| Any comorbidity, n (%) | 28 (22%) | 823 (24%) | 0.72 |

## Table S6. Missing data proportions in each group of socioeconomic deprivation in BRAGGSS

|  | Overall | 20% most deprived | Middle 40% | 40% least deprived |
| --- | --- | --- | --- | --- |
| N | 3459 | 770 | 1256 | 1433 |
| Age | 74 (2.1) | 21 (2.7) | 29 (2.3) | 24 (1.7) |
| Sex | 2 (0.1) | 1 (0.1) | 1 (0.1) | 0 (0) |
| RF positive | 583 (16.9) | 122 (15.8) | 209 (16.6) | 252 (17.6) |
| Age at diagnosis | 67 (1.9) | 22 (2.9) | 18 (1.4) | 27 (1.9) |
| BMI | 857 (24.8) | 167 (21.7) | 351 (28.0) | 339 (23.7) |
| Ever smoked | 705 (20.4) | 132 (17.1) | 256 (20.4) | 317 (22.1) |
| Baseline DAS28 | 243 (7.0) | 57 (7.4) | 95 (7.6) | 91 (6.4) |
| Tender joint count | 152 (4.4) | 41 (5.3) | 60 (4.8) | 51 (3.6) |
| Swollen joint count | 152 (4.4) | 40 (5.2) | 61 (4.9) | 51 (3.6) |
| ESR | 748 (21.6) | 97 (12.6) | 279 (22.2) | 372 (26.0) |
| CRP | 208 (6.0) | 51 (6.6) | 73 (5.8) | 84 (5.9) |
| Patient global VAS | 218 (6.3) | 52 (6.8) | 82 (6.5) | 84 (5.9) |
| HAQ | 593 (17.1) | 169 (22.0) | 208 (16.6) | 216 (15.1) |
| Data presented as number of patients with missing data (percentage). RF, rheumatoid factor. No missing data for “any comorbidity”. | | | | |

## Table S7. Proportion of missing baseline and 6-month DAS28 and components in BRAGGSS.

|  |  | n | % |
| --- | --- | --- | --- |
| baseline | DAS28 | 243 | 7 |
|  | Tender joint count | 152 | 4.4 |
|  | Swollen joint count | 152 | 4.4 |
|  | CRP | 208 | 6 |
|  | Patient global score | 218 | 6.3 |
| 3 months | DAS28 | 714 | 20.6 |
|  | Tender joint count | 596 | 17.2 |
|  | Swollen joint count | 597 | 17.3 |
|  | CRP | 656 | 19 |
|  | Patient global score | 675 | 19.5 |
| DAS28 could be calculated using CRP or ESR as per the recruiting centre. | | | |

## Table S8. Imputed treatment response measures at baseline and 6 months in BRAGGSS.

|  |  | 20% most deprived | Middle 40% | 40% least deprived |
| --- | --- | --- | --- | --- |
| Percentage response at 3 months | Remission | 22.3% | 24.2% | 26.2% |
|  | Low disease activity | 35.3% | 39.8% | 41.1% |
|  | No EULAR response | 23.7% | 22.1% | 19.7% |
|  | Moderate EULAR response | 41.8% | 39.6% | 39.7% |
|  | Good EULAR response | 34.5% | 38.3% | 40.6% |
| Baseline | DAS28, mean | 5.71 | 5.68 | 5.72 |
|  | Tender joint count, mean | 14.4 | 14.6 | 14.6 |
|  | Swollen joint count, mean | 7.7 | 8.2 | 8.8 |
|  | CRP, mean, mg/l | 22.2 | 22.3 | 21.6 |
|  | Patient global, mean | 75.0 | 71.9 | 73.5 |
| 3 months | DAS28, mean | 3.84 | 3.70 | 3.62 |
|  | Tender joint count, mean | 6.5 | 6.1 | 5.8 |
|  | Swollen joint count, mean | 2.8 | 3.0 | 3.2 |
|  | CRP, mean, mg/l | 12.5 | 10.9 | 10.6 |
|  | Patient global, mean | 46.3 | 43.6 | 42 |

## Figure S3. Models comparing 3-month DAS components across groups of socioeconomic deprivation in BRAGGSS.


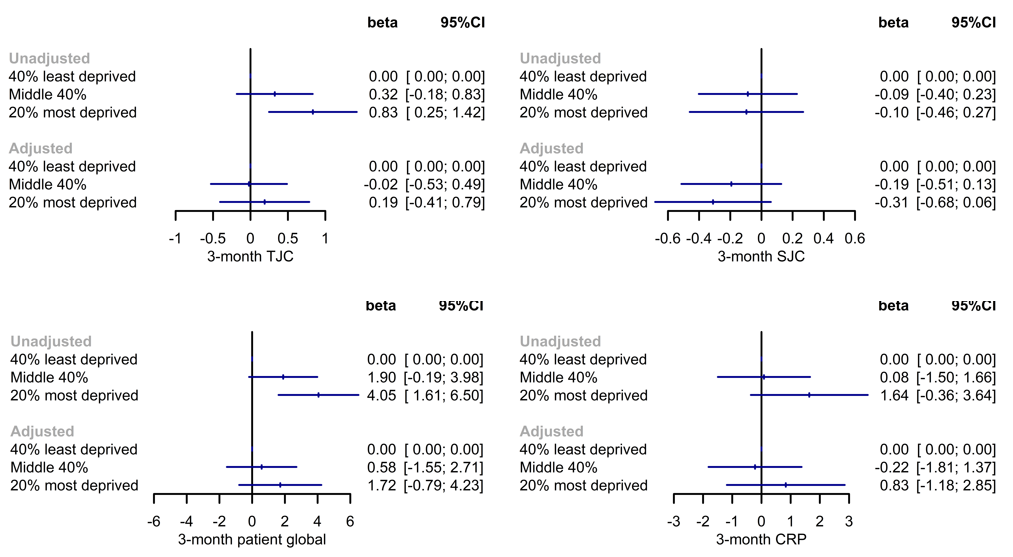

Supplement: kead261_Supplementary_Data [file kead261_supplementary_data.docx]
